# Effects of Multi-Fluorinated Liquid Crystals with High Refractive Index on the Electro-Optical Properties of Polymer-Dispersed Liquid Crystals

**DOI:** 10.3390/ma18071406

**Published:** 2025-03-21

**Authors:** Yunxiao Ren, Wei Hu

**Affiliations:** Institute for Advanced Materials and Technology, University of Science and Technology Beijing, Beijing 100083, China; d202110673@xs.ustb.edu.cn

**Keywords:** polymer dispersed liquid crystal, thiol-ene, viewing angle, refractive index

## Abstract

Polymer-dispersed liquid crystals (PDLCs) are composite materials, in which LCs are dispersed in the form of microdroplets in a polymer matrix. As a composite material, its electro-optical properties are affected by many factors such as molecular structure, composition, and the microstructure of the LCs and polymers. In this work, PDLC films were prepared based on the thiol-ene click reaction, and effects of refractive indexes of polymers and LCs on their electro-optical properties were studied. The refractive indexes of the polymer matrix are adjusted by controlling the content of sulfur element, and those of the LCs are adjusted by adding multi-fluorinated LCs with high refractive index. By regulating the refractive indexes of the polymer matrix and LCs, the maximum transmittance of the film is raised and the viewing angle of the film is also extended. This work could afford some ideas for the directional regulation of the viewing angles and the electro-optical properties of the PDLC film.

## 1. Introduction

A polymer-dispersed liquid crystal (PDLC) film, composed of LCs and a polymer matrix, is an electric-switchable optical film, which has the advantages of high contrast, simple preparation, no polarizer, flexibility, etc. Due to their excellent electro-optical performances, PDLC films can be used in large-size flexible display devices, nonlinear optical materials, electronically controlled smart glasses, selective permeability films, thermal devices, LC gratings, holographic films, and optical switches [1,2,3,4,5,6].

With the development of the PDLC applications, more and more attention has been paid to its durability and viewing angle. PDLC film is a typical composite material: the LCs act as a functional component to offer electro-optic responsiveness and the polymer networks act as matrix materials to offer mechanical support. The mechanical strength and adhesion of the polymer matrix largely determine the stability and service life of PDLC. The matching degree between the refractive indexes of the polymer matrix and the LC molecules determines the open-state transmittance and the viewing angle width of the PDLC [7].

In order to solve these problems, scientists have tried to prepare high-performance PDLCs with different material systems and preparation methods. Polymerization-induced phase separation (PIPS) is the most common PDLC preparation method. The polymerization systems can be divided into three different kinds: (1) epoxy system; (2) acrylate system; and (3) thiol-ene system. Among them, the epoxy system is more of a thermal polymerization, with high energy consumption and low efficiency, while acrylates mostly use more toxic monomers and have poor mechanical properties [8,9,10,11]. In addition, acrylate polymerization is a free-radical homopolymerization reaction, and the polymerization rate is usually very fast and difficult to control, which is not conducive to the fine regulation of PDLC properties. Thiol-ene click chemistry has a wide range of applications as a connection tool in organic synthesis, materials science, molecular biology and biotechnology, polymer synthesis, and other fields because of its multiple advantages [12,13,14,15,16,17]. The polymer matrix based on the thiol-ene system can offer more efficient reaction processes and a wider range of refractive index regulation possibilities [18,19]. The thiol-ene click reaction typically proceeds under mild conditions, characterized by rapid reaction rates and high yields, enabling transformations to be completed in a short time. Moreover, this reaction exhibits high chemical and regioselectivity, allowing for the precise introduction of thiol or alkene functional groups into complex molecular structures. Due to its mild reaction conditions and minimal by-products, the thiol-ene click reaction is considered an environmentally friendly synthetic method and is well-suited for modular synthesis strategies, facilitating the rapid construction of complex molecular architectures [20]. Polyvinylidene fluoride is also a very promising matrix material candidate for electro-optical materials due to its piezoelectric and dielectric properties, which could provide alternative solutions for smart optical applications [21,22].

In PDLC films, LC molecules in the form of micron-sized droplets are dispersed in a polymer matrix, and the LCs are aligned in a spontaneous direction to minimize the free energy [23,24,25]. Because the alignment direction between different droplets is random, the difference between the effective refractive index (*n_eff_*) of the LCs and the refractive index of the polymer (*n_p_*) is large at this time, leading to a macroscopic light scattering of the film. There is a specific relationship between n_eff_ and the ordinary refractive index (*n_o_*) and extraordinary refractive index (*n_e_*) of the LCs:

(1) $$n_{eff}=\frac{n_{o}n_{e}}{\sqrt{n_{e}^{2}{cos}^{2}\alpha +n_{o}^{2}{sin}^{2}\alpha }}$$

where α is the angle between the LC molecule and the incident light [26,27,28]. If the n_o_ of the LCs matches n_p_, the PDLC cell can be completely converted to transparent when sufficient voltage is applied to align all droplets along the vertical direction (assuming that the main LC has positive dielectric anisotropy) [29,30,31]. However, when the refractive indices of the LCs and polymer matrix are poorly matched, the optical performance and viewing angle of the PDLC film is poor [6,32,33,34,35,36,37]. According to Snell’s law, when light travels between two different media, the ratio of the sine of the incidence angle to the sine of the refraction angle is equal to the ratio of the refractive indexes of the two media:

n_1_sinθ_1_ = n_2_sinθ_2_ (2)

where n_1_ and n_2_ are, respectively, the refractive index of the two media; θ_1_ is the incidence angle, that is, the angle when the light enters the first medium; and θ_2_ is the refraction angle, that is, the angle after the light enters the second medium. Obviously, the smaller the refractive index difference between the two interfaces, the less the light is refracted. Therefore, in order to obtain a wide viewing angle PDLC film, the difference between n_e_ and n_p_ should be reduced as much as possible.

Different functional groups have a significant impact on the properties of liquid crystals. The introduction of fluorine-containing groups notably enhances the antioxidant and anti-degradation capabilities of liquid crystal molecules, making them highly stable under strong light and high-temperature conditions. Additionally, fluorine-containing groups effectively reduce the surface energy of liquid crystal molecules, thereby improving their wettability and self-assembly properties. Furthermore, fluorine-containing liquid crystals exhibit low viscosity and short response times, while their high dielectric anisotropy further enhances response speed, making them highly suitable for high-resolution and fast-response electro-optical devices [38].

**Figure 1:**
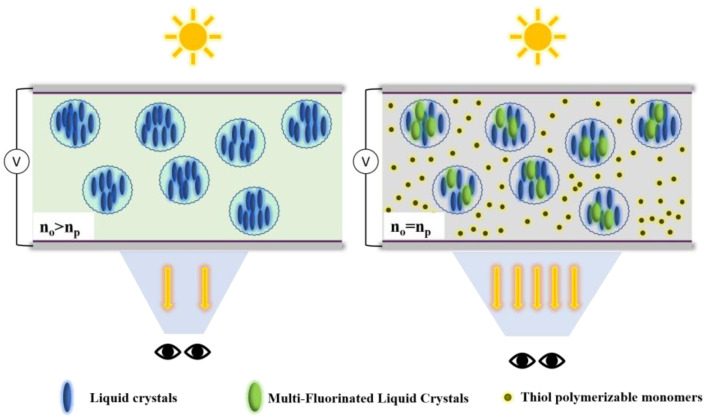
Schematic diagram of the principle of view-angle regulation.

In PDLC, it is very important to match the refractive index of LCs with that of the polymer matrix. In order to make the refractive index of the polymer (*n_p_*) equal to the ordinary refractive index of the LCs (*n_o_*), the refractive index of the LC mixture can be adjusted by adding monomers with different refractive indexes to the LC mixture and different heteroatoms can be introduced into the polymer chain to adjust the refractive index of the polymer. In this work, we adopted a high-refractive-index liquid crystal monomer containing fluorine to adjust the refractive index of the LC mixture, and adjusted the content of the S element in the polymer molecular chain to change the refractive index of the polymer matrix. This bidirectional adjustment strategy makes the performance adjustment of PDLC more precise and efficient, achieving better performance. Herein, the PDLC film viewing angle was controlled by adjusting the refractive index of the polymer and LC. Schematic diagram of the principle of view-angle regulation is shown in Figure 1. It not only improves the maximum transmittance of the film, but it also optimizes the defects of the PDLC film perspective to a certain extent. This provides severe useful research on the electro-optical properties and viewing angles of PDLC films.

## 2. Materials and Methods

**Figure 2:**
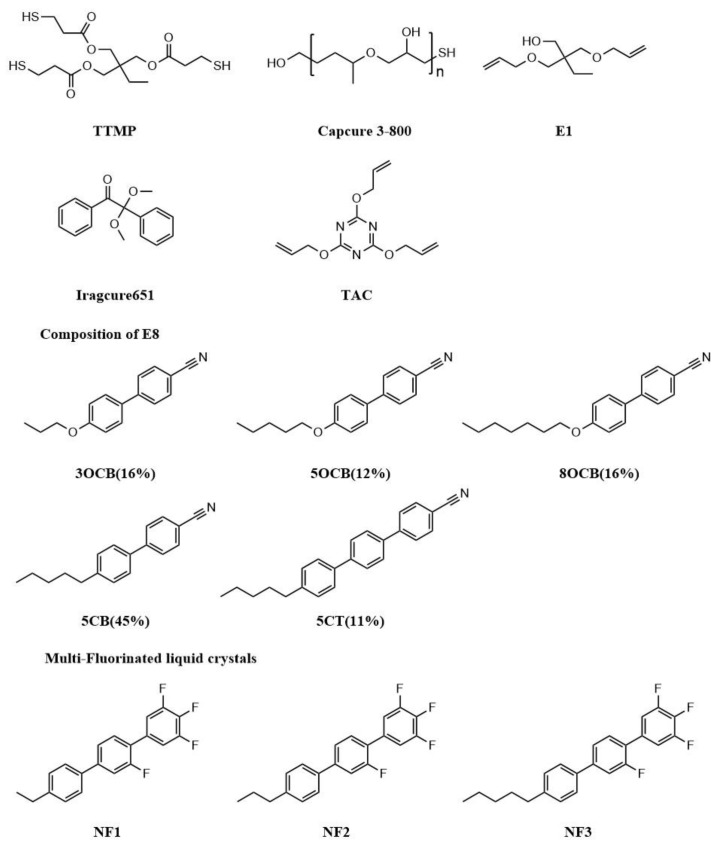
Chemical structures of the materials used in this study.

The thiol monomer trimethylolpropanetris(3-mercaptopropionate) (TTMP) was gifted by TCI (Shanghai, China) Development Co., Ltd. The ene monomer 2,2-bis(allyloxymethyl)-1-butanol (E1) was obtained from Macklin Co., Ltd., Shanghai, China. Triallyl cyanurate (TAC), used as the crosslinking monomer, was provided from Aladdin Industrial Inc., Shanghai, China. Capcure 3–800 was purchased from Jiadida (Shenzhen, China) Co., Ltd. Iragcure 651 was purchased from Heowns Biochem Technologies, Tianjin, China. LC Monomers (NF1 (T_NI_ = 364 K), NF2 (T_CI_ = 339 K) and NF3 (T_CI_ = 332 K)) were purchased from Beijing Jinxunyangguang Electronic Materials Technology Co., Ltd., Beijing, China. The nematic LC E8 (T_NI_ =346 K, Δn = 0.248, n_o_ = 1.525) was purchased from Jiangsu Hecheng Display Technology Co., Ltd., Nanjing, China. The chemical structures of components are shown in Figure 2; these materials were used as received without further purification.

Precursor mixtures composed of LCs, polymerizable monomer, and photoinitiator Iragcure 651 were mixed well, according to the ratios in Table 1. All the samples were filled into non-oriented LC cells with a thickness of 20 μm and irradiated under UV light at 5 mW/cm^2^ for 300 s. The effective area of the LC cells is 40 × 40 mm^2^.

The prepared PDLC film samples were soaked in a bottle with cyclohexane for 2 weeks, and the cyclohexane was changed periodically to remove the LC molecules. The soaked samples were dried in a vacuum oven for 24 h. After gold spraying on the surface, the samples could be observed by scanning electron microscopy (SEM, HITACHI S-4800, Tokyo, Japan).

The electro-optical characteristics of all PDLC samples were tested at room temperature using a LC device parameter tester (LCT-5016C, Changchun Liancheng Instrument Co., Ltd., Changchun, China). A halogen laser (λ = 560.0 nm) was used as the incident light source. The test voltage is increased by 700 mV per second when using the LCT-5016C device for turn-on response time (t_on_) testing. The transmittance of blank cells was normalized to 100%.

The liquid crystal monomer NF was measured using a Perkin Elmer Pyris 6 differential scanning calorimeter (DSC, Waltham, MA, USA). The sample was placed in an aluminum crucible, pressed into a tablet, and then tested under the protection of high-purity nitrogen gas. The heating and cooling scan rate was set at 5 °C/min.

The PDLC film viewing angle was tested using an angular light intensity distribution tester. The prepared PDLC film sample was placed on the carrier table. The distance between the sample and the planar light source was 40 mm. The distance between the light detector and the sample was 20 cm. The sample and the light source were rotated simultaneously. The rotation angle was from −90° to 90°. The corresponding light intensity was recorded in real-time. The effective size of all the samples tested was 40 × 40 mm^2^.

## 3. Results

### 3.1. Effects of Polymer Refractive Index on Electro-Optical Properties and Viewing Angle of PDLC Films

**Figure 3:**
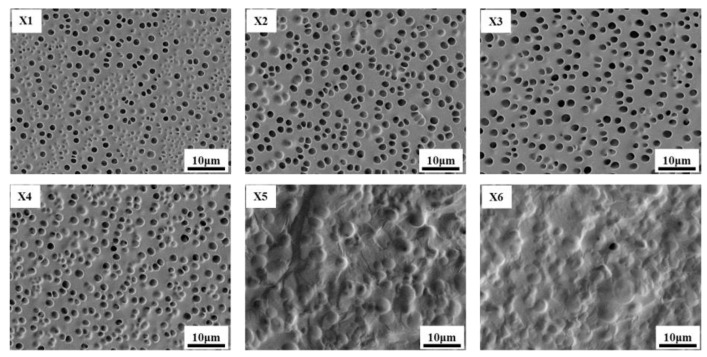
SEM micrographs of the polymer network in Samples X1–X6.

Figure 3 shows the SEM micrographs of Samples X1–X6 prepared by PIPS. As the concentration of Capcure 3–800 increases, it is observed that the average sizes of LC droplets tend to increase. It should be noted that, although the pore sizes of the polymer mesh of Samples X2, X3 and X5 are compared separately, their differences are not obvious. However, from the overall perspective, the pore size of Sample X1 is undoubtedly the smallest, and the mesh sizes of X4 and X5 are much larger than those of the previous groups of samples, so the overall trend of mesh size increase can be confirmed from the Samples X1–X5. This is because that Capcure 3–800 has a much smaller mercaptan value compared with the TTMP. Therefore, the increase of Capcure 3–800 in the mixture led to a decrease in the sulfhydryl content, which certainly resulted in a decrease in the polymerization rate. As a result, the decrease in the crosslink density of the polymer and the increase in LC droplet size occur in the composite films.

**Figure 4:**
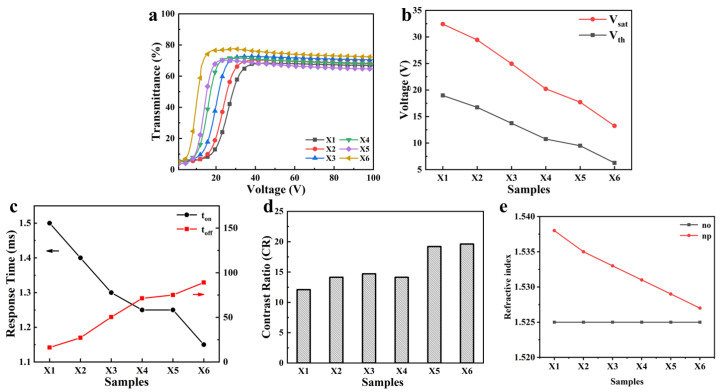
Electrical-optical properties of PDLC films X1–X6. (**a**) Voltage-dependent transmittance of Samples X1–X6; (**b**) Threshold voltage (Vth) and saturation voltage (V_sat_) of Samples X1–X6; (**c**) Turn-on response time (t_on_), black curve corresponds to the left ordinate (black arrow points) and turn-off response time (t_off_), red curve corresponds to the left ordinate (red arrow points); (**d**) Contrast ratio (CR) of Samples X1–X6; (**e**) Refractive index of Samples X1–X6.

Figure 4a shows the electro-optical properties of PDLC films X1–X6. With the increase of Capcure 3–800 content, the scattered-state and open-state transmittance of the samples both show an increasing trend. This could be explained by the increase in the polymer mesh size leading to the decrease of the scattering centers in the PDLC films. The driving voltage decreased with the increase of Capcure 3–800 content, which was also due to the increase in the polymer network mesh size (Figure 4b). Sample X6 was the minimum on-state response time, which was related to its larger polymer mesh size (Figure 4c). Meanwhile, the anchoring force of the polymer interface to the LC molecules was reduced and the rate of LC molecule reorientation was accelerated. On the contrary, the off-state response time became relatively long. The contrast ratios of all the samples are indicated in Figure 4d, and Sample X6 has the highest contrast ratio, which is related to its higher transmittance of the transparent state. As shown in Figure 4e, it can be distinctly observed that the refractive indices of the LCs and polymer are becoming closer and closer from Samples X1 to X6, with the polymer refractive index reducing due to the reduction of the large refractive index element S content in the polymer chain.

**Figure 5:**
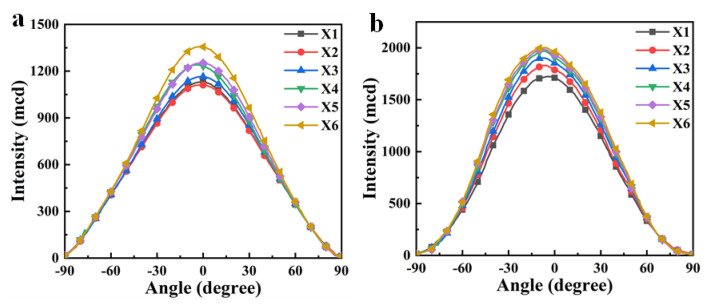
Viewing angle test of X1–X6 at different voltages: (**a**) 0 V; (**b**) 30 V.

In addition, the sulfhydryl functional group (S atom) content decreases with the increase in Capcure 3–800 content in the system. Therefore, the refractive index of the polymer matrix n_p_ decreases due to the content of sulfur-containing groups with large molar refractive index reduced, as shown in Table 1. With the decrease in n_p_ from Samples X1 to X6, the difference values between n_o_ and n_p_ become smaller. Therefore, Sample X6 has higher optical transmittance and a wider viewing angle (Figure 5).

### 3.2. Effects of LC Refractive Index on Electro-Optical Properties and Viewing Angle of PDLC Films

Multi-fluorinated LC monomers with high refractive index NF1, NF2, and NF3 were mixed uniformly according to the mass ratio of 1:1:1, called NF. The NF and the nematic phase LC E8 were mixed according to a certain ratio, and the LC parameters were tested and recorded in Table 1. Because all the NF LCs have terphenyl structural units, they exhibit high extraordinary refractive indexes n_e_. However, they also have multi-fluorinated substituent groups on the two sides of the benzene rings; the ordinary refractive index increases more significantly with the increase of molecular width. Therefore, the birefringences (Δn = n_e_ − n_o_) of the LC mixtures become larger with the increasing contents of the NF LCs.

**Figure 6:**
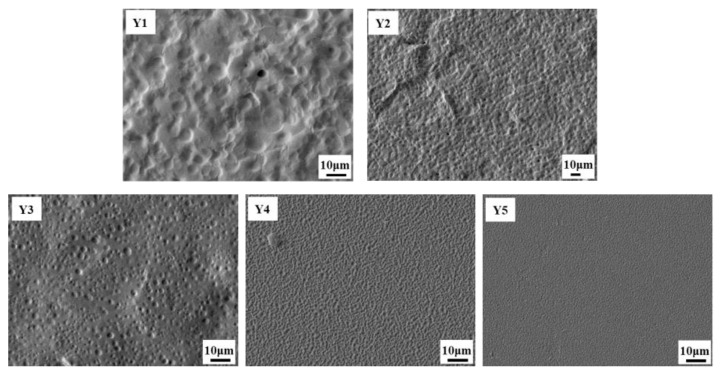
SEM micrographs of the polymer network in Samples Y1–Y5.

Figure 6 shows the SEM images of the polymer network morphology of Samples Y1–Y5. With the increase in NF LCs content, it is found that the polymer network mesh size becomes smaller, especially in Samples Y4–Y5. This may be caused by the increased viscosity of the system, which reduces the diffusion rate of liquid crystal droplets during phase separation. In addition, the mesh formed is smoother with the fluorine-containing monomer content due to the lubrication action of fluorinated organic matter.

**Figure 7:**
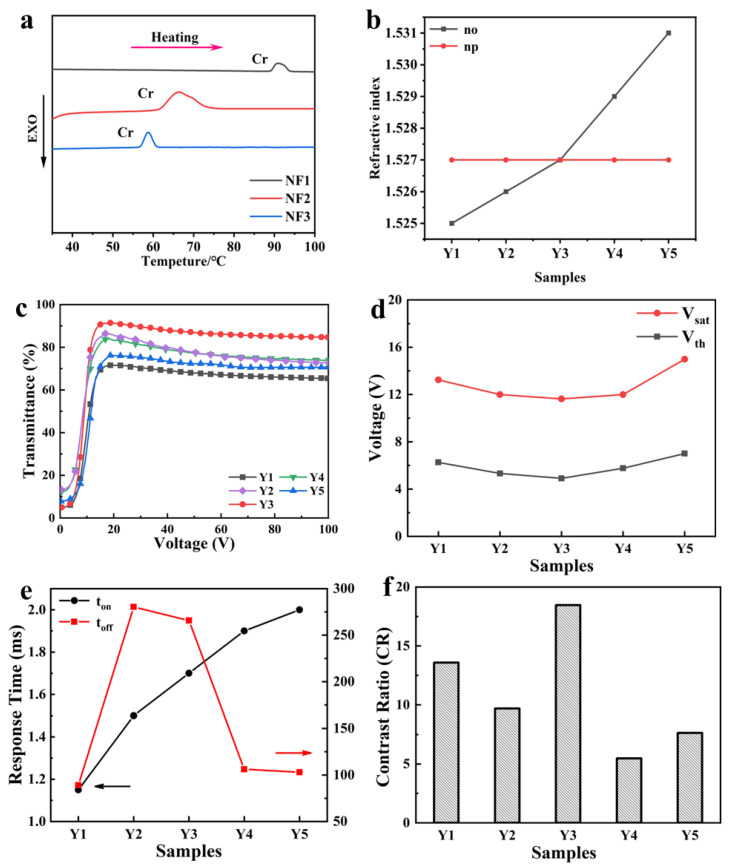
The electrical and optical properties of PDLC films Y1–Y5. (**a**) The DSC curve of NF monomer; (**b**) The refractive index of Samples Y1–Y5. (**c**) Voltage-dependent transmittance of Samples Y1–Y5; (**d**) Threshold voltage (Vth) and saturation voltage (V_sat_) of Samples Y1–Y5; (**e**) Turn-on response time (t_on_), black curve corresponds to the left ordinate (black arrow points) and turn-off response time (t_off_), red curve corresponds to the left ordinate (red arrow points); (**f**) Contrast ratio (CR) of Y1–Y5.

The differential scanning calorimetry (DSC) curves of multi-fluorinated liquid crystals with high refractive index NF1, NF2 and NF3 are shown in Figure 7a; they all have relatively high melting points. Although a small amount of doping of a single NF monomer also has some impact on the viewing angle; however, increasing the doping amount of a single monomer can easily lead to crystal precipitation. Therefore, we chose to dope three structurally similar NF monomers to increase the total doping amount, achieving better performance. As shown in Figure 7b, the ordinary refractive index n_o_ of LC mixture increases with the NF doping amount increasing from Samples Y1 to Y5. And the n_o_ of LC mixture in Sample Y3 is equal to the refractive index of the polymer n_p_, obtaining the best refractive index matching degree. However, with the further increase in the refractive index n_o_ of the LC mixture in Samples Y4 and Y5, this refractive index difference appears again and becomes larger.

Figure 7c–f shows the electro-optical performances of the Samples Y1–Y5. Figure 7c shows that the open-state transmission of the samples rises first and then decreases as the mixed NF LC content increases. From Figure 7d, it can be seen that the voltage decreases first and then increases. Despite the mesh diameters of Samples Y1–Y3 being similar, the smoother mesh can reduce the anchoring force of the polymer interface, so the saturation voltage of PDLC films decreases first. On the other hand, due to the sharp decrease in the mesh sizes of Samples Y4–Y5 matrix networks, the anchoring effect of the polymer network on the LC becomes larger and the anchoring force becomes the main influencing factor; then, the voltage shows an increasing trend. Figure 7e shows the variation pattern of open-state response time and off-state response time of the samples. The decreasing sample mesh sizes and the increasing LC viscosity both lead to the slowing down of the LC reorientation, so the open-state response time gradually increases. After the electric field is withdrawn, the increasing viscosity leads to the slowing down of the LC recovery, and the anchoring effect results in the off-state response time increasing first and then decreasing. In Figure 7f, since the liquid crystal refractive index of sample Y3 matches best with the refractive index of the polymer matrix, it exhibits the highest transmittance in the on-state, and, accordingly, its contrast ratio is the best among all the samples.

**Figure 8:**
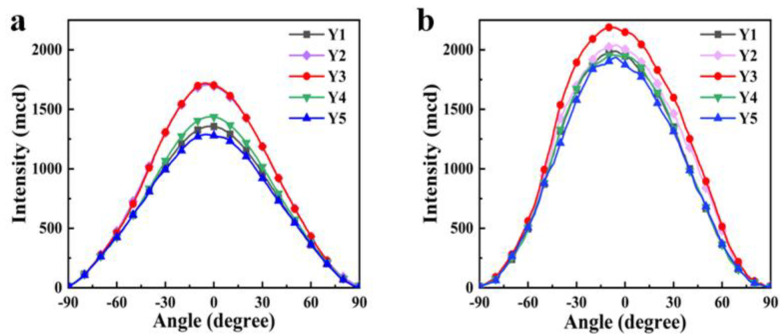
The viewing angle of Y1–Y5 at different voltages: (**a**) 0 V; (**b**) 15 V.

Because the driving voltage of Samples Y1–Y5 significantly decreased, their viewing angle tests were conducted under a 15 V applied electric field condition (Figure 8). Samples Y2 and Y3 exhibit a wider viewing angle; this is due to the best match between the refractive index of the polymer matrix n_p_ and the n_o_ of the LC mixture being present in these two groups of samples. From Table 1, it can be seen that both the n_o_ and n_p_ of Sample Y3 are the same value, 1.527, meaning that the incident light penetrating from the polymer matrix to the LC droplet would not cause significant scattering at the open-state. To sum up, in order to obtain PDLC films with wide viewing angles and excellent electro-optical properties, it is an effective strategy to match the refractive indexes of LCs and polymers by adjusting the composition and ratio of the material system.

## 4. Conclusions

In this work, the PDLC films were prepared by the PIPS method based on the thiol-ene click reaction. Wide viewing angles and excellent electro-optical properties were obtained by adjusting the composition and ratio of the material system. The sulfur-containing polymerizable monomers with large molar refractive index were used to modulate the refractive index of the polymer matrix; multi-fluorinated LC molecules with high refractive index were used to adjust the birefringence of LC mixtures. When the refractive index of the liquid crystal and the polymer is best matched, the PDLC film with the best electro-optical properties and the widest viewing angle is obtained. We hope that this work could afford some inspiration for researchers to improve the electro-optical performance and regulate the viewing angles of PDLC films.

## Figures and Tables

**Table 1:** Compositions of the samples.

Sample ID	TTMP/Capcure 3–800/TAC/E1 (wt%)	E8/NF	n_o_	Δn	n_p_	η (mPa·s)
X1	27.50/0/13.25/13.25	45.00/0	1.525	0.248	1.538	49.50
X2	24.75/2.75/13.25/13.25	45.00/0	1.525	0.248	1.535	49.50
X3	22.00/5.50/13.25/13.25	45.00/0	1.525	0.248	1.533	49.50
X4	19.25/8.25/13.25/13.25	45.00/0	1.525	0.248	1.531	49.50
X5	16.50/11.0013.25/13.25	45.00/0	1.525	0.248	1.529	49.50
X6	13.75/13.7513.25/13.25	45.00/0	1.525	0.248	1.527	49.50
Y1	13.75/13.7513.25/13.25	45.00/0	1.525	0.248	1.527	49.50
Y2	13.75/13.7513.25/13.25	40.50/4.50	1.526	0.210	1.527	50.00
Y3	13.75/13.7513.25/13.25	36.00/9.00	1.527	0.208	1.527	54.60
Y4	13.75/13.7513.25/13.25	31.50/13.50	1.529	0.203	1.527	57.60
Y5	13.75/13.7513.25/13.25	27.00/18.00	1.531	0.200	1.527	63.60

Note: X6 and Y1 are samples of the same formulation.

## Data Availability

The original contributions presented in this study are included in the article. Further inquiries can be directed to the corresponding author.

## Abbreviations

The following abbreviations are used in this manuscript:

PDLC	Polymer-Dispersed Liquid Crystal
LC	Liquid Crystal
PIPS	Polymerization-Induced Phase Separation
